# EnforceSNN: Enabling resilient and energy-efficient spiking neural network inference considering approximate DRAMs for embedded systems

**DOI:** 10.3389/fnins.2022.937782

**Published:** 2022-08-10

**Authors:** Rachmad Vidya Wicaksana Putra, Muhammad Abdullah Hanif, Muhammad Shafique

**Affiliations:** ^1^Embedded Computing Systems, Institute of Computer Engineering, Technische Universität Wien, Vienna, Austria; ^2^eBrain Lab, Division of Engineering, New York University Abu Dhabi (NYUAD), Abu Dhabi, United Arab Emirates

**Keywords:** spiking neural networks, high performance, energy efficiency, approximate computing, approximate DRAM, DRAM errors, error tolerance, resilience

## Abstract

Spiking Neural Networks (SNNs) have shown capabilities of achieving high accuracy under unsupervised settings and low operational power/energy due to their bio-plausible computations. Previous studies identified that DRAM-based off-chip memory accesses dominate the energy consumption of SNN processing. However, state-of-the-art works do not optimize the DRAM energy-per-access, thereby hindering the SNN-based systems from achieving further energy efficiency gains. To substantially reduce the DRAM energy-per-access, an effective solution is to decrease the DRAM supply voltage, but it may lead to errors in DRAM cells (i.e., so-called *approximate DRAM*). Toward this, we propose *EnforceSNN*, a novel design framework that provides a solution for resilient and energy-efficient SNN inference using reduced-voltage DRAM for embedded systems. The key mechanisms of our EnforceSNN are: (1) employing quantized weights to reduce the DRAM access energy; (2) devising an efficient DRAM mapping policy to minimize the DRAM energy-per-access; (3) analyzing the SNN error tolerance to understand its accuracy profile considering different bit error rate (BER) values; (4) leveraging the information for developing an efficient fault-aware training (FAT) that considers different BER values and bit error locations in DRAM to improve the SNN error tolerance; and (5) developing an algorithm to select the SNN model that offers good trade-offs among accuracy, memory, and energy consumption. The experimental results show that our EnforceSNN maintains the accuracy (i.e., no accuracy loss for *BER* ≤ 10^−3^) as compared to the baseline SNN with accurate DRAM while achieving up to 84.9% of DRAM energy saving and up to 4.1x speed-up of DRAM data throughput across different network sizes.

## 1. Introduction

**Figure 1:**
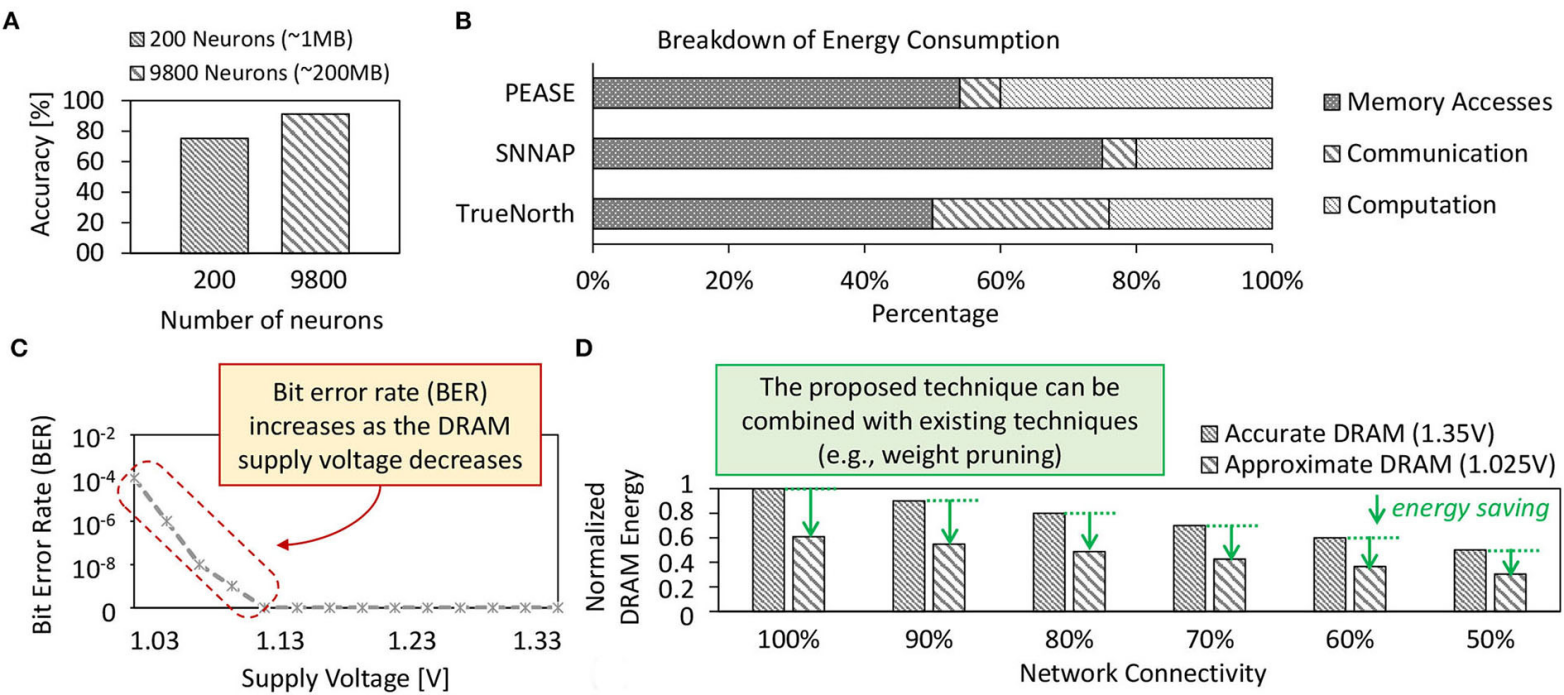
**(A)** Accuracy profiles of small-sized and large-sized SNN models on the MNIST dataset which are obtained from our experiments. **(B)** Breakdown of the energy consumption of SNN processing on different SNN hardware platforms, i.e., PEASE (Roy et al., 2017), SNNAP (Sen et al., 2017), and TrueNorth (Akopyan et al., 2015); adapted from studies in Krithivasan et al. (2019). **(C)** Bit error rate (BER) of approximate DRAM and its respective supply voltage *V*_*supply*_; adapted from studies in Chang et al. (2017). **(D)** The estimated DRAM energy savings achieved by our technique when combined with the weight pruning across different rates of network connectivity (i.e., synaptic connections) for a network with 4900 excitatory neurons. The results are obtained from experiments using the LPDDR3-1600 4Gb DRAM configuration and the DRAMPower simulator (Chandrasekar, 2014).

Spiking neural networks (SNNs) have demonstrated the potential of obtaining high accuracy under unsupervised settings and low operational energy due to their bio-plausible spike-based computations (Putra and Shafique, 2020). A larger SNN model is usually favorable as it offers higher accuracy than the smaller ones, as shown by our experimental results in Figure 1A. Here, the 1MB-sized network achieves only 75%, while the 200MB-sized network achieves 92% accuracy for the MNIST dataset. This MNIST dataset is a set of training and test images for handwritten digits 0–9 (Lecun et al., 1998). On the other hand, most of the SNN hardware platforms have relatively small on-chip memory, e.g., less than 100MB (Roy et al., 2017; Sen et al., 2017; Frenkel et al., 2019a,b). Therefore, running an SNN model with a larger size than the on-chip memory of SNN hardware platforms will require intensive access to the off-chip memory. Previous studies show that single access to the off-chip memory (i.e., DRAM) incurs significantly higher energy consumption than single access to the on-chip memory (i.e., SRAM) (Sze et al., 2017; Putra et al., 2021b). Moreover, previous study also identified that memory access dominate the energy consumption of SNN processing, incurring 50–75% of the total system energy across different SNN hardware platforms, as shown in Figure 1B. The reason is that DRAM access energy is significantly higher than other SNN operations (e.g., neuron operations) (Krithivasan et al., 2019). This problem is even more critical for AI applications with stringent constraints (e.g., low-cost embedded devices with a small on-chip memory size) (Shafique et al., 2021) since it leads to even more intensive DRAM accesses. Consequently, this problem hinders SNN-based embedded systems from obtaining further efficiency gains.

**Targeted Research Problem:** *How can we substantially decrease the DRAM access energy for the SNN inference, while maintaining accuracy*. The solution to this problem will enable efficient SNN inference for energy–constrained embedded devices and their applications for Edge-AI and Smart CPS. *Edge-AI* is the system that runs Artificial Intelligence (AI) algorithms on resource- and energy-constrained computing devices at the edge of the network, i.e., close to the source of data (Shi et al., 2016; Satyanarayanan, 2017; Yu et al., 2018; Chen and Ran, 2019; Liu et al., 2019; Cao et al., 2020). Meanwhile, *Smart CPS (Cyber-Physical System)* is the system that includes the interacting networks of computational components (e.g., computation and storage devices), physical components (e.g., sensors and actuators), and human users (Chattopadhyay et al., 2017; Griffor et al., 2017; Kriebel et al., 2018; Shafique et al., 2018).

### 1.1. State-of-the-art and their limitations

To decrease the energy consumption of SNN inference, state-of-the-art works have developed different techniques, which can be loosely classified as the following.

- **Reduction of the SNN operations** through approximate neuron operations (Sen et al., 2017), weight pruning (Rathi et al., 2019), and neuron removal (Putra and Shafique, 2020). These techniques decrease the number of DRAM accesses for the corresponding model parameters.
- **Quantization** by reducing the range of representable values for SNN parameters (e.g., weights) (Rathi et al., 2019; Putra and Shafique, 2020; Putra and Shafique, 2021a). These techniques reduce the amount of SNN parameters (e.g., weights) to be stored in and fetched from DRAM.

**Limitations:** These state-of-the-art works mainly aim at reducing the number of DRAM accesses, but do not optimize the DRAM energy-per-access and do not employ approximations in DRAM that provide an additional knob for obtaining high energy efficiency. Therefore, optimization gains offered by these works are sub-optimal, hindering the SNN inference systems from achieving the full potential of DRAM energy savings. Therefore, the effective optimization should jointly minimize the DRAM energy-per-access and the number of DRAM accesses, by leveraging the approximation in DRAM to expose the full energy-saving potential, while overcoming the negative impact of the approximation-induced errors (i.e., bit-flips in DRAM cells). Figure 1C shows the approximation-induced error rates in DRAM.

To address these limitations, *we employ approximate DRAM (i.e., DRAM with reduced supply voltage) with efficient DRAM data mapping policy and fault-aware training to substantially reduce the DRAM access energy in SNN inference systems while preserving their accuracy*. Moreover, our proposed technique can also be combined with state-of-the-art techniques to further improve the energy efficiency of SNN inference. For example, Figure 1D shows the estimated DRAM energy savings achieved by our technique when combined with the weight pruning. To highlight the potential of reduced-voltage approximate DRAM, we perform an experimental case study in the following section.

### 1.2. Motivational case study and key challenges

**Figure 2:**
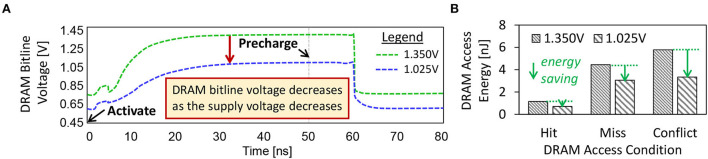
**(A)** The dynamics of *V*_*bitline*_ under different *V*_*supply*_ values. **(B)** DRAM access energy for a row buffer hit, a row buffer miss, and a row buffer conflict, under different *V*_*supply*_ values.

In the case study, we aim at studying (1) the dynamics of DRAM bitline voltage (*V*_*bitline*_) for both the accurate and approximate DRAM settings, and (2) the DRAM access energy for different access conditions (including a row buffer hit, miss, or conflict). Note, *V*_*bitline*_ is defined as the voltage measured in each DRAM bitline when a DRAM supply voltage (*V*_*supply*_) is applied, as shown in Figures 2A, **4C**. Further details on the dynamics of *V*_*bitline*_ are provided in Section 2.2.2. For DRAM access conditions, a row buffer hit means that the requested data has been loaded in the DRAM row buffer, thus the data can be accessed without additional DRAM operations. Meanwhile, a row buffers miss or conflict needs to open the requested DRAM row before the data can be loaded into the row buffer and then accessed. Further information on the DRAM access conditions is discussed in Section 2.2.1.

For the experimental setup, we employ the DRAM circuit model from the study of Chang et al. (2017) and the SPICE simulator to study the dynamics of *V*_*bitline*_. The accurate DRAM operates at 1.35V of the supply voltage (*V*_*supply*_), while the approximate one operates at 1.025V. Further details on the experimental setup are discussed in Section 5. Furthermore, we consider the LPDDR3-1600 4Gb DRAM configuration as it is representative of the low-power DRAM types for embedded systems. We employ the DRAMPower simulator to estimate the DRAM access energy because it has been validated against real measurements (Chandrasekar, 2014) and has been widely used in the computer architecture communities. Figure 2 presents the experimental results, from which we make the following key observations.

- The *V*_*bitline*_ decreases as the *V*_*supply*_ decreases, hence forcing the DRAM cells to operate under lower reliability as the *weak cells* may fail to hold the correct bits. *Weak cells* are DRAM cells that fail when the DRAM parameters (e.g., voltage, timing) are reduced (Chang et al., 2017; Kim et al., 2018).
- The reduced-voltage DRAM decreases the DRAM energy-per-access across different access conditions, i.e., by up to 42% of energy reduction for each access.
- The row buffer hit has lower energy consumption than the row buffer miss or conflict. Moreover, row buffer hit also incurs less latency than the row buffer miss or conflict (Putra et al., 2020; Putra et al., 2021b). Therefore, the row buffer hit should be exploited to optimize the DRAM latency and energy.

Although employing the approximate DRAM can substantially decrease the DRAM energy-per-access, it also decreases the DRAM reliability since the bit errors increase when the *V*_*supply*_ decreases, as shown in Figure 1C. These errors may degrade the accuracy of SNN inference since they can change the weight values in DRAM, which then deteriorates the neuron behavior.

**Associated Research Challenge:** *How to achieve low DRAM access energy for SNN inference using approximate DRAM, while minimizing their negative impact on the accuracy*.

### 1.3. Our novel contributions

To overcome the above research challenges, we propose **the EnforceSNN framework**, which enables resilient and energy-efficient SNNs considering approximate DRAMs (i.e., reduced-voltage DRAMs) for embedded systems. Based on the best of our knowledge, it is the first effort that employs approximate DRAM for improving the energy efficiency of SNN inference, while enhancing their error tolerance against bit errors in DRAM. Our EnforceSNN framework employs the following key steps.

1. **Employing weight quantization** to reduce the memory footprint for SNN weights and the number of DRAM accesses for SNN inference, thereby optimizing the DRAM access energy.
2. **Devising an efficient DRAM data mapping** to maximize row buffer hits for optimizing the DRAM energy-per-access while considering BER in DRAM.
3. **Analyzing the SNN error tolerance** to understand the SNN accuracy profile under different DRAM supply voltage and different BER values.
4. **Improving the SNN error tolerance** by developing and employing efficient fault-aware training (FAT) that considers SNN accuracy profile and bit error locations in DRAM.
5. **Devising an algorithm to select the SNN model** that offers good trade-offs among accuracy, memory, and energy consumption from the given model candidates using the proposed reward function.

**Key Results:** We evaluate the EnforceSNN framework for (1) classification accuracy using PyTorch-based simulations (Hazan et al., 2018) on a multi-GPU machine considering the MNIST and Fashion MNIST datasets^1^, and (2) DRAM access energy using DRAMPower (Chandrasekar, 2014). We perform an epoch of unsupervised learning (60K experiments) for each retraining process considering each combination of the SNN model, workload, and training BER; then perform inference (10K experiments) for each combination of the SNN model, workload, and testing BER. The experimental results indicate that our EnforceSNN reduces the DRAM access energy by up to 84.9% and improves the speed-up up to 4.1x while maintaining the accuracy (no accuracy loss) across different network sizes for *BER* ≤ 10^−3^.

## 2. Background

### 2.1. Spiking neural networks

**Figure 3:**
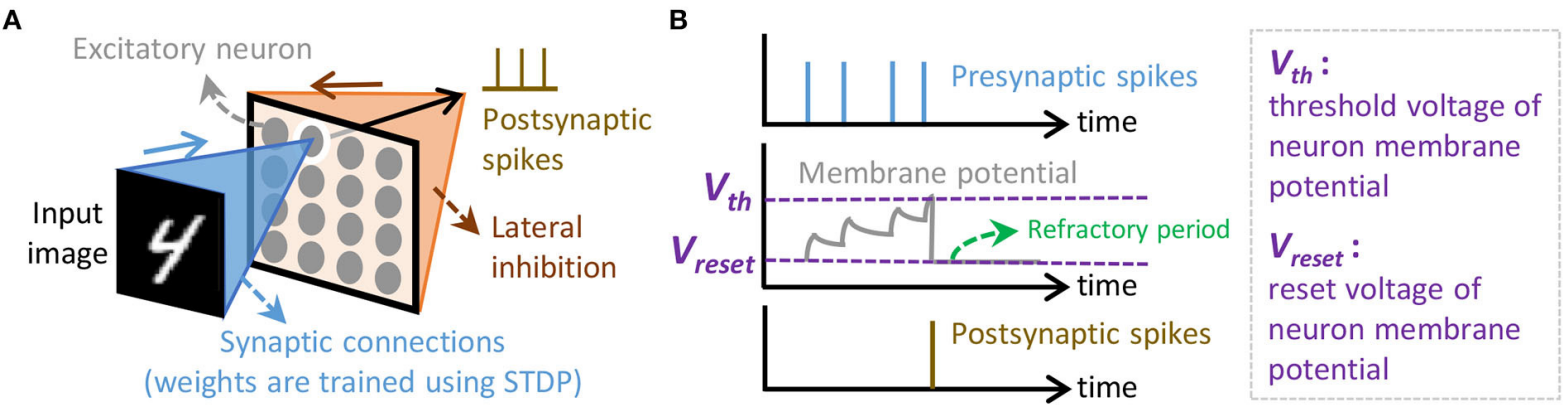
**(A)** The SNN architecture considered in this study, which is adapted from Putra and Shafique (2020). **(B)** The neuronal dynamics of the Leaky Integrate-and-Fire (LIF) neuron model.

Spiking Neural Networks are the neural network models that employ bio-plausible computations and use the sequences of spikes (i.e., *spike trains*) for conveying information. These spikes are encoded using a specific spike coding. Several spike coding schemes have been proposed in the literature (Gautrais and Thorpe, 1998; Thorpe and Gautrais, 1998; Kayser et al., 2009; Park et al., 2019; Park et al., 2020). Here, we use rate coding as it has been used widely and offers robustness for diverse learning rules (Diehl and Cook, 2015; Putra et al., 2021a). For the learning rule, we use the spike-timing-dependent plasticity (STDP), as it has been widely used by previous works (Diehl and Cook, 2015; Hazan et al., 2018; Saunders et al., 2018; Putra and Shafique, 2021b). In an SNN model, neurons and synapses are connected in a specific architecture (Pfeiffer and Pfeil, 2018; Mozafari et al., 2019; Tavanaei et al., 2019). Here, we consider a fully-connected architecture as it supports unsupervised learning scenarios; refer to Figure 3A. In this architecture, each input pixel is connected to all (excitatory) neurons, and the output of each neuron is connected to other neurons for providing inhibition. For the neuron model, we use the Leaky Integrate-and-Fire (LIF) as it provides bio-plausible neuronal dynamics with low computational complexity (Izhikevich, 2004; Putra et al., 2022); refer to Figure 3B.

### 2.2. Approximate DRAM

#### 2.2.1. DRAM fundamentals

**Figure 4:**
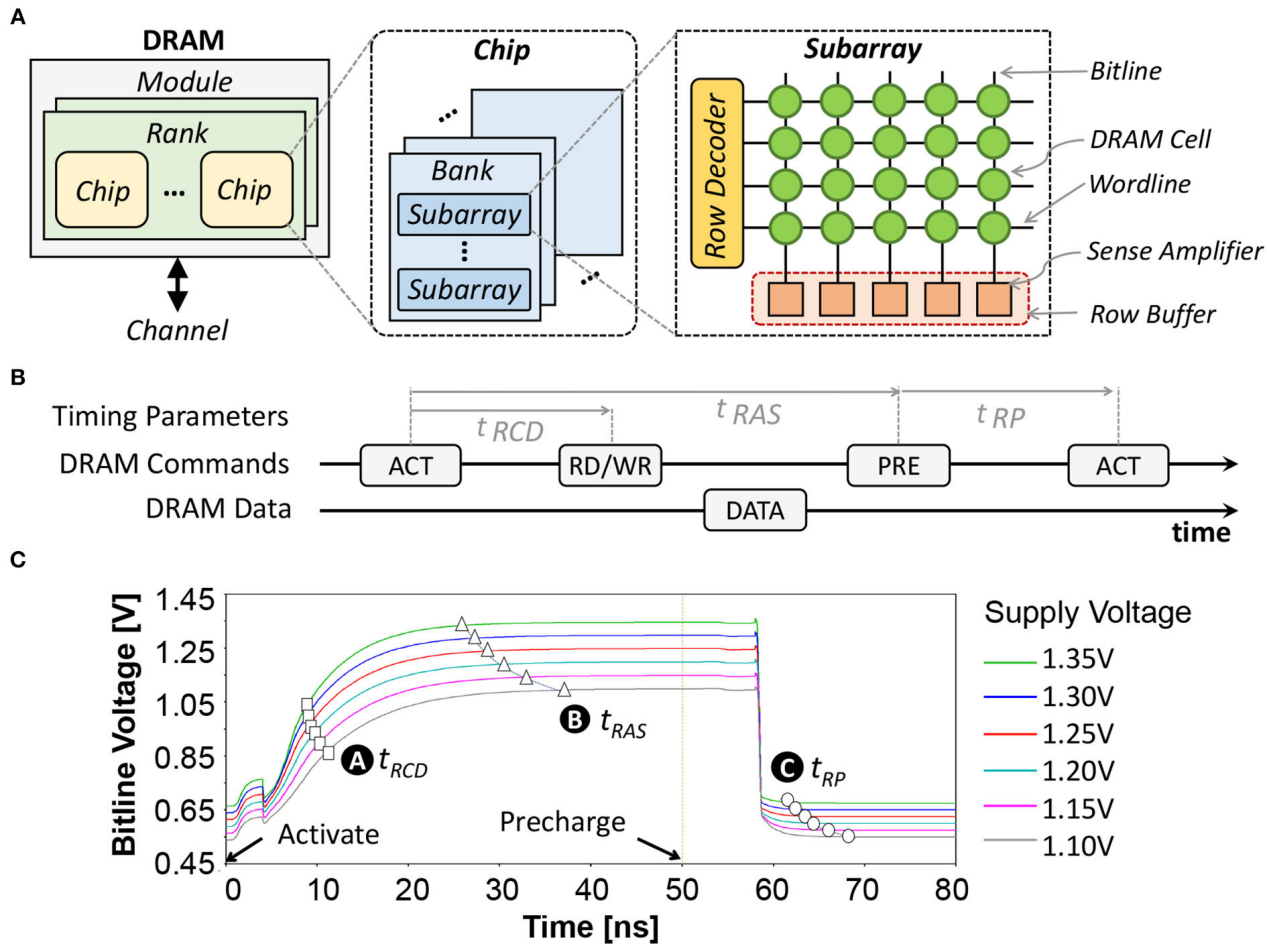
**(A)** The internal organization of commodity DRAM. **(B)** Diagram of the DRAM commands (i.e., ACT, RW or WR, and PRE) and the DRAM timing parameters (i.e., *t*_*RCD*_, *t*_*RAS*_, and *t*_*RP*_). **(C)** The dynamics of the DRAM bitline voltage *V*_*bitline*_ and the respective timing parameters.

The organization of a DRAM consists of channel, module, rank, chip, bank, subarray, row, and column (Putra et al., 2020; Olgun et al., 2021); refer to Figure 4A. A single DRAM request can access data from multiple DRAM chips within the same rank in parallel. In each DRAM chip, the request is routed to a specific bank, row, and column address. When the activation (ACT) command is issued, the requested row is opened and its data are copied to the row buffer. If the read (RD) command is issued, data in the row buffer can be read. If the write (WR) command is issued, data in the row buffer can be replaced with the new one. In each DRAM request, there are different possible access conditions, i.e., a row buffer hit, miss, and conflict (Ghose et al., 2019). A row buffer hit refers to a condition when the requested row is activated and its data are already in the row buffer. Hence, the data can be accessed directly without additional operation. Otherwise, the requested row is still closed, and the condition is either a row buffer miss or conflict. A row buffer miss is defined if there is no activated row when a request happens, thus the requested row should be activated before accessing the data. Meanwhile, a row buffer conflict is defined when the requested row is still closed, but the row buffer is occupied by another activated row. Hence, the activated row should be closed using the precharging (PRE) command, before activating the requested row using the activation (ACT) command. Figure 4B illustrates the DRAM commands (i.e., ACT, RD or WR, PRE) and their timing parameters (i.e., *t*_*RCD*_: row address to column address delay, *t*_*RAS*_: row active time, and *t*_*RP*_: row precharge time).

#### 2.2.2. Reduced-voltage DRAM

We perform extensive experiments using the SPICE simulator and the DRAM circuit model from Chang et al. (2017) while considering different supply voltage (*V*_*supply*_) values, to characterize the parameters of reduced-voltage DRAM: including the bitline voltage (*V*_*bitline*_) and the respective timing parameters (i.e., *t*_*RP*_, *t*_*RCD*_, and *t*_*RAS*_). The experimental results are presented in Figure 4C, and the obtained parameters are used for further DRAM energy estimation. The ready-to-access voltage is defined as the condition when *V*_*bitline*_ reaches 75% of *V*_*supply*_, which represents the minimum *t*_*RCD*_ for reliable DRAM operations, as shown by 🅐. The ready-to-precharge voltage is defined as the condition when *V*_*bitline*_ reaches 98% of *V*_*supply*_, which represents the minimum *t*_*RAS*_ for reliable DRAM operations, as shown by 🅑. Meanwhile, the ready-to-activate voltage is defined as the condition when the *V*_*bitline*_ is within 2% of *V*_*supply*_/2, which represents the minimum *t*_*RP*_ for reliable DRAM operations, as shown by 🅒.

## 3. Error modeling for approximate DRAM

The study of Koppula et al. (2019) has proposed four error models that closely fit the real reduced-voltage-based approximate DRAMs as the following. **Error Model-0**: the bit errors follow a *uniform random distribution* across a DRAM bank; **Error Model-1**: the bit errors follow a *vertical distribution* across the bitlines of a DRAM bank; **Error Model-2**: the bit errors follow a *horizontal distribution* across the wordlines of a DRAM bank; and **Error Model-3**: the bit errors follow a *uniform random distribution* that depends on the content of the DRAM cells. In this study, we employ the **DRAM Error Model-0**, due to the following reasons: (1) it produces errors with high similarity to the real reduced-voltage-based approximate DRAM by using the percentage of weak cells and the error probability in any weak cell; (2) it offers a reasonable approximation of other error models, including the approximation of (a) errors across bitlines similar to Error Model-1, (b) errors across wordlines similar to Error Model-2, and (c) uniform random distribution similar to Error Model-3; and (3) it provides fast error injection by software. Previous work (Koppula et al., 2019) also employed the DRAM Error Model-0 majorly due to similar reasons.

## 4. EnforceSNN framework

### 4.1. Overview

**Figure 5:**
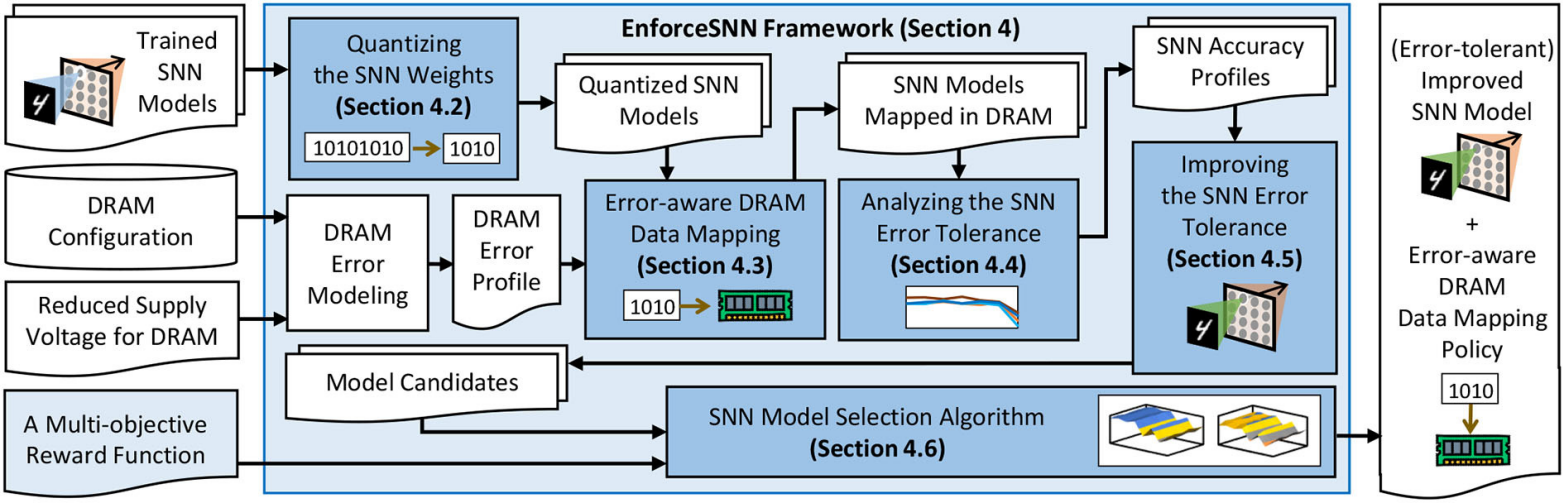
The EnforceSNN framework and its novel mechanisms (highlighted in blue boxes).

Our EnforceSNN framework employs several key steps as shown in Figure 5. First, we *quantize the SNN weights* to reduce memory footprint and DRAM access energy (Section 4.2). Second, we devise *an error-aware DRAM data mapping policy* to optimize the DRAM energy-per-access (Section 4.3). These optimizations contribute to 4.1x inference speed-up and 84.9% DRAM access energy saving (Section 6). Then, we *analyze the SNN error tolerance* to understand the accuracy profile of SNN inference under different BER values (Section 4.4). We leverage this information to *develop an efficient FAT technique that improves the SNN error tolerance* (Section 4.5). These fault tolerance techniques contribute to 2.71x retraining speed-up without accuracy loss for *BER* ≤ 10^−3^ (Section 6). We also *develop an SNN model selection algorithm* to find a model that provides good trade-offs among accuracy, memory, and energy consumption (Section 4.6). Details of these mechanisms are explained in the following subsections.

### 4.2. Quantizing the SNN weights

**Figure 6:**
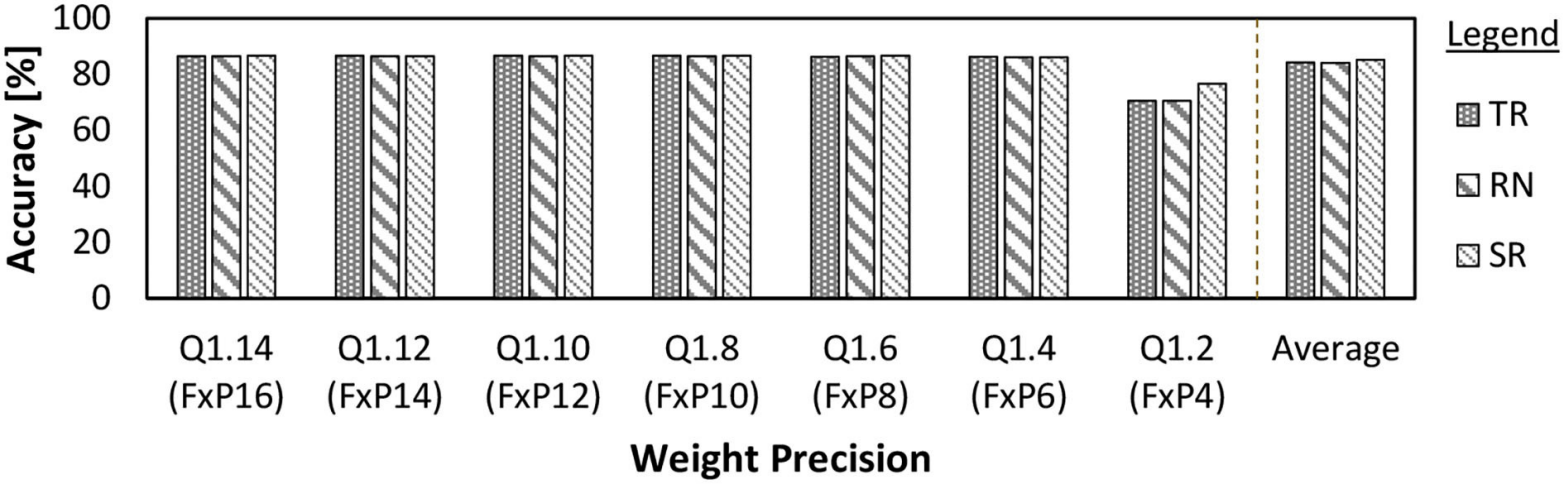
The accuracy of a 900-neuron network on the MNIST across different precision levels and rounding schemes, i.e., truncation (TR), round to the nearest (RN), and stochastic rounding (SR). Here, we follow the definition of TR, RN, and SR from the study of Hopkins et al. (2020). Note that the fixed-point format *Qi*.*f* can also be represented as FxP(1+*i*+*f*). These results show that employing TR on top of the FxP8 precision leads to competitive accuracy as compared to other rounding schemes (i.e., RN and SR).

*We perform weight quantization to substantially reduce the memory footprint and the number of DRAM accesses, which leads to DRAM energy saving*. The reason is that quantization is a prominent technique for reducing the memory footprint of neural networks without decreasing the accuracy significantly (Gupta et al., 2015; Micikevicius et al., 2018). Moreover, it is the first effort to study and exploit SNN weight quantization considering approximation errors in DRAM, thereby providing new insights as compared to previous studies on SNN weight quantization. Our weight quantization considers the fixed-point format which can be represented as *Qi*.*f*. It denotes 1 sign bit, *i* integer bits, and *f* fractional bits, and follows the 2's complement format (i.e., signed *Qi*.*f*). Here, the range of representable values is [−2^*i*^, 2^*i*^−2^−*f*^] with the precision of ϵ = 2^−*f*^. We select the “signed *Qi*.*f*” format to show that our EnforceSNN framework provides a generic solution with high applicability for different variants of bio-plausible learning rules (e.g., STDP variants) which may lead to positive or negative weight values (Rahimi Azghadi et al., 2014; Diehl and Cook, 2015). To do this, we perform a fixed-point quantization to the trained SNN weights using a specific rounding scheme, such as truncation, round to the nearest, or stochastic rounding. For a study case, we select truncation as it provides competitive accuracy with low computational complexity (Putra and Shafique, 2021a, 2022a,b). To illustrate this, we evaluate the impact of different rounding schemes on the accuracy through an experimental case study, and the results are shown in Figure 6. Truncation (TR) keeps the *f* bits and removes the other fractional bits. Therefore, the output fixed-point for the given real number *x* with *Qi*.*f* format is defined as *TR*(*x, Qi*.*f*) = ⌊*x*⌋. In our SNN model, we employ the pair-based weight-dependent STDP learning rule from the study of Diehl and Cook (2015) that bounds each weight value (*w*) within the defined range, i.e., *w* = [0, 1]. Consequently, applying the truncation to the weights will round the value down. In this study, we consider an 8-bit fixed-point with “signed *Q*1.6” and 2's complement format (i.e., 1 sign bit, 1 integer bit, and 6 fractional bits), since it provides high accuracy for SNNs under unsupervised learning scenarios (Putra and Shafique, 2021a). Note, we can also employ the “unsigned *Qi*.*f*” format without sign bit to represent the 8-bit non-negative weights (i.e., 1 integer bit and 7 fractional bits) in the EnforceSNN if desired. For both “signed *Qi*.*f*” and “unsigned *Qi*.*f*” formats, 1 bit for the integer part is required for representing the maximum possible weight value (i.e., *w* = 1).

**Quantization Steps:** We quantize only the weights through the simulated quantization approach, which represents the weight values under fixed-point format, and performing computations under floating-point format (Jacob et al., 2018; Krishnamoorthi, 2018; Gholami et al., 2021; van Baalen et al., 2022). To perform quantization, we convert the weight values from 32-bit floating-point format (FP32) to 8-bit fixed-point format (signed *Q*1.6) by constructing their 8-bit binary representations under 32-bit integer format (INT32), thereby conveniently performing bit-wise modification and rounding operation while considering the sign and the rounding scheme (i.e., truncation). Afterward, we convert the quantized weight values (INT32) to FP32 format through casting and then normalize the values by 2^*f*^. Hence, the 8-bit binary representations of quantized weight values are saved in FP32 and can be used in the FP32-based arithmetic computations.

**DRAM Error Injection:** *If there is no DRAM error*, the quantization steps are performed and the quantized weight values (in FP32) can be used for computations in SNN processing. *If DRAM errors exist*, the quantization steps are performed while considering the DRAM error injection. These errors are injected into the 8-bit binary representations of quantized weights (in INT32) under a specific DRAM data mapping policy. Afterward, we convert the binary representations of quantized weights (in INT32) to FP32 format, so that the quantized weight values can be used for computations in SNN processing.

### 4.3. Error-aware DRAM data mapping policy

**Figure 7:**
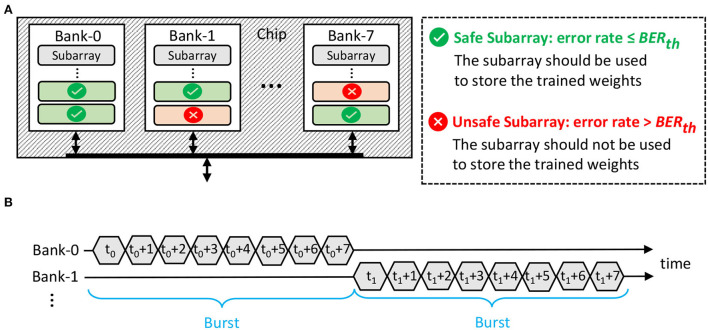
**(A)** Our proposed DRAM mapping policy, leveraging subarray-level granularity. **(B)** The timing diagram of the DRAM multi-bank burst feature.

It is important to map the SNN model properly in DRAM to ensure that (1) the weights are minimally affected by errors in DRAM so that the accuracy is maintained, and (2) the DRAM energy-per-access is optimized. Toward this, *we devise and employ an error-aware DRAM mapping policy to place the SNN weights in DRAM, while optimizing the DRAM energy-per-access*. The proposed DRAM mapping policy is illustrated in Figure 7A, and its key ideas are explained in the following.

**Figure 8:**
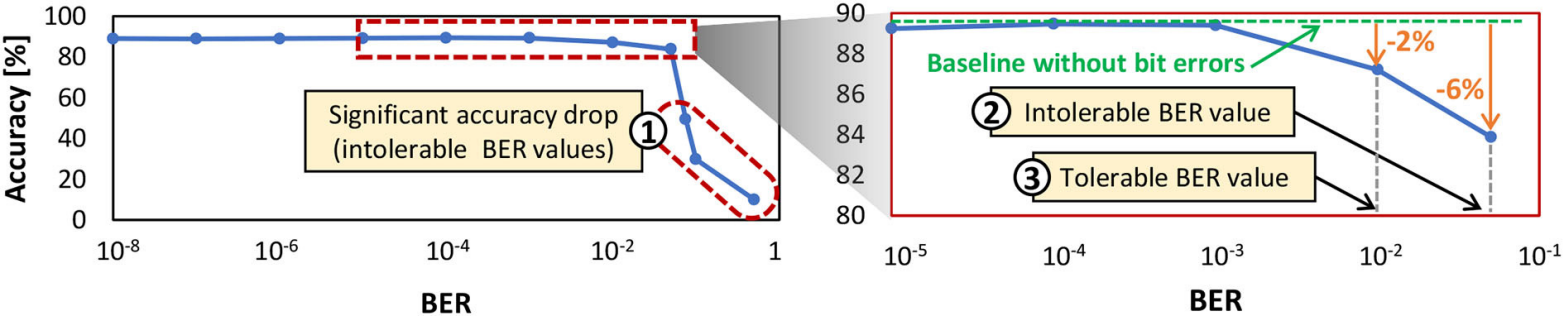
The test accuracy profile of a 900-neuron network across different BER values shows the tolerable and intolerable BER. This network has a fully-connected architecture like in Figure 3A where each input pixel is connected to all neurons, and the output of each neuron is connected to other neurons for performing inhibition, thereby providing competition among neurons to correctly recognize the input class.

1. The weights are mapped in the appropriate DRAM part (e.g., chip, bank, or subarray), whose error rate meets the BER requirement, i.e., ≤ the maximum tolerable BER (*BER*_*th*_). Here, we consider the subarray-level granularity for data mapping, since it allows us to exploit the following features.
  - *The multi-bank burst feature*, which is available in commodity DRAM, can be employed to increase the throughput. Its timing diagram is illustrated in Figure 7B.
  - *The subarray-level parallelism*, which is available in novel DRAM architectures (Kim et al., 2012), can also be employed to increase the throughput.
We determine *BER*_*th*_ through experiments that investigate the accuracy profile of a network across different BER values. Figure 8 shows the experimental results for a 900-neuron network. If the accuracy scores are significantly lower than the baseline accuracy without bit errors (i.e., >3% accuracy degradation), we refer to the respective BER values as *intolerable BER*, as shown in ① and ② in Figure 8. Otherwise, we define them as *tolerable BER*. For instance, ③ in Figure 8 shows an accuracy that is associated with a tolerable BER. Based on this discussion, we define $BER_{th}=10^{-2}$.
2. The weights are mapped in a way to maximize the row buffer hits for optimizing the DRAM energy-per-access while exploiting the multi-bank burst feature for maximizing the data throughput. The reason is that a row buffer hit incurs the lowest DRAM access energy than other access conditions (i.e., a row buffer miss or conflict), as suggested by our experimental results in Figure 2B.

To efficiently implement the above ideas, we devise Algorithm 1 with the following key steps. First, we identify the subarrays whose error rate ≤ *BER*_*th*_ and refer to them as *the safe subarrays*, which should be used for storing the weights. Otherwise, we refer to the subarrays whose error rate > *BER*_*th*_ as *the unsafe subarrays*, which should not be used for storing the weights. This step is represented in line 7 of Algorithm 1. Second, to maximize the row buffer hits and exploit the multi-bank burst feature, the data mapping in each DRAM chip should follow the following policy (represented in lines 3-8 of Algorithm 1).

- **Step-1:** Assume that we consider mapping data in a target subarray of the target bank with the following initial indices, i.e., *subarray_index = i, bank_index = j*.
- **Step-2:** If the target subarray is a safe subarray, then we prioritize mapping the data in different columns of the same row for maximizing row buffer hits. Otherwise, this subarray is not utilized and we move to another target subarray in a different bank (*subarray_index = i, bank_index += 1*). Then, we perform **Step-2** again. If all columns in the same row across all banks are filled or unavailable, then we move to another subarray in the initial bank (*subarray_index += 1, bank_index = j*) to exploit subarray-level parallelism, if applicable.
- **Step-3:** In the target subarray, the remaining data are mapped in the same fashion as **Step-2**. When all columns in the same row of all safe subarrays across all banks are filled, then the remaining data are placed in a different row of the initial target subarray and bank (*subarray_index = i, bank_index = j*). Afterward, we perform **Step-2** to **Step-3** again until all data are mapped in a DRAM chip. If some data remain but there are no available spaces in a DRAM chip, then we move to **Step-4**.
- **Step-4:** The remaining data are mapped using **Step-1** to **Step-3** in different DRAM chips, ranks, and channels, respectively if applicable.

**Algorithm 1:** **The proposed DRAM mapping policy**.


**INPUT: (1)** DRAM (*DRAM*): number of channel (*n*_*ch*_), number of rank-per-channel (*n*_*ra*_), number of chip-per-rank (*n*_*cp*_), number of bank-per-chip (*n*_*ba*_), number of subarray-per-bank (*n*_*su*_), number of row-per-subarray (*n*_*ro*_), number of column-per-row (*n*_*co*_);
**(2)** Bit error rate (BER): BER of a subarray (*BER*_*subarray*), maximum tolerable BER (*BER*_*th*_);
**(3)** Data (*data*);
**OUTPUT:** DRAM (*DRAM*);
**BEGIN**
**Process**:
1: **for** *ch* = 0 to (*n*_*ch*_−1) **do**
2: **for** *ra* = 0 to (*n*_*ra*_−1) **do**
3: **for** *cp* = 0 to (*n*_*cp*_−1) **do**
4: **for** *ro* = 0 to (*n*_*ro*_−1) **do**
5: **for** *su* = 0 to (*n*_*su*_−1) **do**
6: **for** *ba* = 0 to (*n*_*ba*_−1) **do**
7: **if** *BER*_*subarray*[*ch, ra, cp, ba, su*] ≤ *BER*_*th*_ **then**
8: **for** *co* = 0 to (*n*_*co*_−1) **do**
9: *DRAM*[*ch, ra, cp, ba, su, ro, co*]←*data*;
10: **end for**
11: **end if**
12: **end for**
13: **end for**
14: **end for**
15: **end for**
16: **end for**
17: **end for**
18: **return** *DRAM*;
**END**

### 4.4. Analyzing the SNN error tolerance

**Figure 9:**
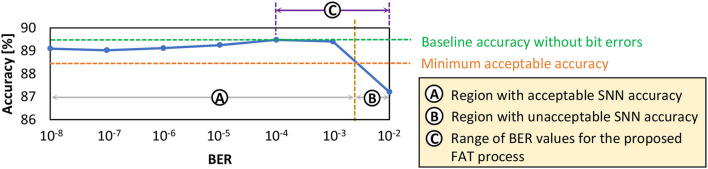
The test accuracy profile of a 900-neuron network shows the region with acceptable accuracy, the region with unacceptable accuracy, and the range of BER values for the proposed fault-aware training. Note that this network is the same as the one in Figure 8. Here, the observation focuses on the BER values that can be considered in the retraining process, thereby having a smaller range than the one in Figure 8.

Previous discussion highlights that bit errors in the SNN weights can degrade the accuracy, as they change the weight values and deviate the neuron behavior from the correct classification. Therefore, SNN error tolerance should be improved, so that the SNN model can achieve high accuracy even in the presence of a high error rate. To effectively enhance the SNN error tolerance, it is important to understand the SNN accuracy profile under DRAM errors. Toward this, *our EnforceSNN analyzes the accuracy profile of the SNN model considering the data mapping pattern in DRAM and different BER values*. We observe that the accuracy profile typically has acceptable accuracy (i.e., within 1% accuracy degradation from the baseline without errors) when BER is low and has notable accuracy degradation when BER is high. Therefore, we classify the accuracy profile into two regions: Ⓐ a region with acceptable accuracy, and Ⓑ a region with unacceptable accuracy, as shown in Figure 9. These insights will be leveraged for developing an efficient enhancement technique for improving the SNN error tolerance in Section 4.5.

### 4.5. Improving the SNN error tolerance

*Our EnforceSNN enhances the SNN error tolerance through the fault-aware training (FAT) technique that incorporates the error profile of the approximate DRAM*. We consider efficiently performing FAT for minimizing training time, energy consumption, and carbon emission during the retraining process (Strubell et al., 2019, 2020), by conducting a small yet effective number of iterations for the retraining process, while avoiding *accuracy collapse*. Accuracy collapse is defined as a significant accuracy degradation due to training divergence that is caused by introducing high BER immediately at the beginning of the retraining process (Koppula et al., 2019). The proposed FAT technique has the following key steps, which are also presented in Algorithm 2.

- **Step-1:** We define the range of BER values that will be incorporated in the training process to make the SNN model adaptable to DRAM errors, as shown by region-Ⓒ in Figure 9. We incorporate (1) BER values in region-Ⓐ that are close to the region-Ⓑ, and (2) all BER values in the region-Ⓑ, in the training process. Specifically, we consider the two highest BER values in region-Ⓐ in the training process to make the model adapt to high fault rates safely, without suffering from significant accuracy degradation (i.e., accuracy collapse) with less training time.
- **Step-2:** The bit errors in DRAM are generated for different BER values (which correspond to different *V*_*supply*_ values), based on the DRAM error model-0 that follows a uniform random distribution across a DRAM bank.
- **Step-3:** The generated bit errors are then injected into the DRAM cell locations, and the weight bits in these locations are flipped. In this step, we consider the proposed DRAM data mapping discussed in Section 4.3 for maximizing the row buffer hits and exploiting the multi-bank burst feature.
- **Step-4:** Afterward, we include the generated bit errors in the retraining by incrementally increasing the BER from the minimum rate to a maximum one following the defined range of BER values from **Step-1**. We increase the BER value after each epoch of retraining by a defined ratio (e.g., 10x of the previous error rate). In this manner, the SNN model is gradually trained to tolerate DRAM errors from the defined lowest rate to the maximum one, thereby carefully improving the SNN error tolerance.

**Algorithm 2:** **The proposed FAT technique**.


**INPUT: (1)** Baseline pre-trained SNN: model (*model*_0_), accuracy (*model*_0_.*acc*);
**(2)** DRAM error model (*DRAMerr*);
**(3)** BER for retraining: error rates (*BER*), number of error rates (*N*_*BER*_);
**(4)** Training dataset: samples (*S*_*train*_), number of samples (*N*_*train*_); **(5)** Test dataset: samples (*S*_*test*_), number of samples (*N*_*test*_);
**OUTPUT: (1)** Improved SNN: model (*model*_1_), accuracy (*model*_1_.*acc*);
**BEGIN**
**Initialization**:
1: *model*_*temp*_ = *model*_0_;
2: *model*_1_ = *model*_0_;
3: *model*_1_.*acc* = 0; **Process**:
4: **for** *i* = 0 to (*N*_*BER*_−1) **do**
5: *error*_*map* = *DRAMerr*(*BER*[*i*]); // error generation
6: inject *error*_*map* into *model*_*temp*_; // error injection
7: **for** *r* = 0 to (*N*_*train*_−1) **do**
8: train *model*_*temp*_ with *S*_*train*_[*r*]; // train
9: **end for**
10: **for** *s* = 0 to (*N*_*test*_−1) **do**
11: test *model*_*temp*_ with *S*_*test*_[*s*]; // test
12: **end for**
13: **if** *model*_*temp*_.*acc*>*model*_1_.*acc* **then**
14: *model*_1_ = *model*_*temp*_;
15: *model*_1_.*acc* = *model*_*temp*_.*acc*;
16: **end if**
17: **end for**
18: **return** *model*_1_;
**END**

### 4.6. Algorithm for SNN model selection

From the previous steps, we may get different sizes of error-tolerant SNN models as potential solutions for the given embedded applications. Therefore, we need to consider design trade-offs to select the most appropriate model for the given accuracy, memory, and energy constraints. Toward this, *we propose an algorithm to quantify the trade-off benefits of the SNN model candidates using our proposed reward function, and then select the one with the highest benefit*. The idea of our multi-objective reward function (*R*) is to prioritize the model that has high accuracy, small memory, and low energy consumption. The reward *R* is defined as the resultant between the accuracy with the memory and energy consumption, as expressed in Equation 1. In this equation, *acc*_*x*_ denotes the accuracy of the investigated SNN model (*x*). *m*_*norm*_ denotes the normalized memory, which is defined as the ratio between the memory footprint of the investigated model (*mem*_*x*_) and the floating-point model (*mem*_*fp*_); refer to Equation 2. The memory footprint of the model is estimated by leveraging the number of weights (*N*_*wgh*_) and the corresponding bit-width (*BW*_*wgh*_); refer to Equation 3. Meanwhile, *E*_*norm*_ denotes the normalized energy consumption, which is defined as the ratio between the DRAM access energy of the approximate DRAM (*E*_*DRAM*_*approx*_) and the accurate one (*E*_*DRAM*_*accurate*_) for the investigated model; refer to Equation 4. To define the significance of memory and energy consumption with respect to the accuracy when calculating *R*, we employ μ and ε as the adjustable trade-off variables for memory and energy consumption, respectively. Here, μ and ε are the non-negative real numbers.


(1) $$\begin{array}{r} R\left(acc_{x},m_{norm},E_{norm}\right)=acc_{x}-\left(\mu \cdot m_{norm}+\varepsilon \cdot E_{norm}\right) \end{array}$$

(2) $$\begin{array}{r} m_{norm}=\frac{mem_{x}}{mem_{fp}} \end{array}$$

(3) $$\begin{array}{r} mem=N_{wgh}\cdot BW_{wgh} \end{array}$$

(4) $$\begin{array}{r} \begin{array}{c} E_{norm}=\frac{E_{DRAM\text{\_}approx}}{E_{DRAM\text{\_}accurate}} \end{array} \end{array}$$

## 5. Evaluation methodology

**Figure 10:**
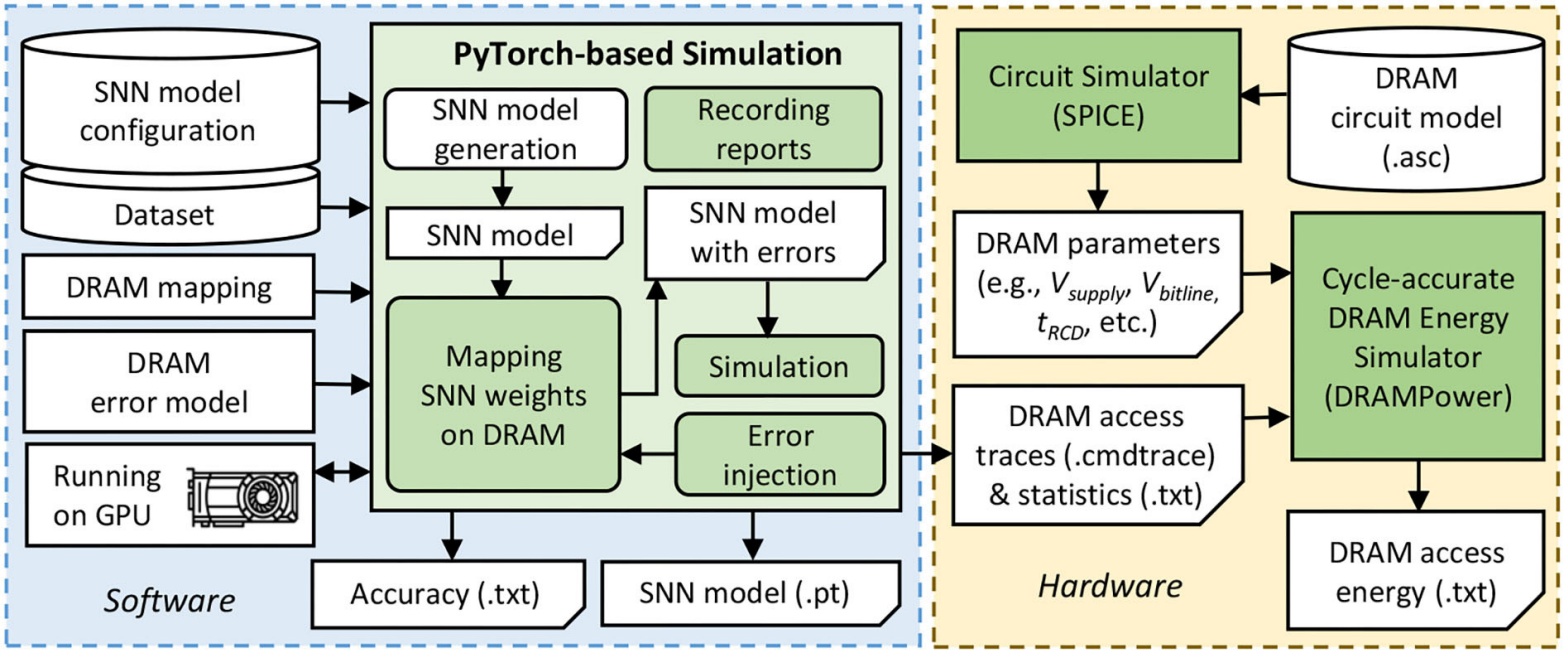
Our experimental setup and tools flow.

Figure 10 shows the experimental setup and tools flow for evaluating our EnforceSNN framework, which is explained in the following.

**Accuracy Evaluation:** We employ PyTorch-based simulations (Hazan et al., 2018) with 32-bit floating-point (FP32) and 8-bit fixed-point precision (i.e., FxP8 with “signed *Q*1.6” and “unsigned *Q*1.7” formats) that run on a multi-GPU machine, i.e., Nvidia GeForce RTX 2080 Ti. For network architecture, we consider the fully-connected network with a different number of excitatory neurons, which are referred to as N-*i* for conciseness with *i* denoting the number of excitatory neurons. We use the rate coding for converting each input pixel into a spike train and use the MNIST and Fashion MNIST datasets. For comparison partners, we use the SNN model which is pre-trained without considering DRAM errors as the baseline. We perform an epoch of STDP-based unsupervised learning through 60K experiments for each retraining process considering each combination of the SNN model, dataset, and training BER. Afterward, we perform inference through 10K experiments for each combination of the SNN model, dataset, and testing BER.

**DRAM Error Generation and Injection:** First, we generate bit errors based on the DRAM error model-0, and inject them into the DRAM cell locations, while considering the data mapping policy in DRAM. Afterward, the weight bits that are stored in the DRAM cell locations with errors, will be flipped. For the baseline data mapping, we place the weight bits in the subsequent address in a DRAM bank to maximize the DRAM burst feature, and if a DRAM bank is filled, then the weight bits are mapped in a different bank of the same DRAM chip. Meanwhile, we use the proposed DRAM mapping in Algorithm 1 for our EnforceSNN.

**DRAM Energy Evaluation:** We use the DRAM circuit model from the work of Chang et al. (2017) and the SPICE simulator to extract the DRAM operational parameters (e.g., *V*_*supply*_, *V*_*bitline*_, *t*_*RCD*_, *t*_*RAS*_, *t*_*RP*_) while considering the configuration of LPDDR3-1600 4Gb DRAM which is representative for the main memory of embedded systems. The accurate DRAM operates with 1.35V of *V*_*supply*_, while the approximate one operates with 1.025V-1.325V of *V*_*supply*_. Afterward, we use the state-of-the-art cycle-accurate DRAMPower (Chandrasekar, 2014) that incorporates the DRAM access traces and statistics as well as the extracted DRAM parameters for estimating the DRAM access energy.

## 6. Results and discussion

### 6.1. Improvements of the SNN error tolerance

**Figure 11:**
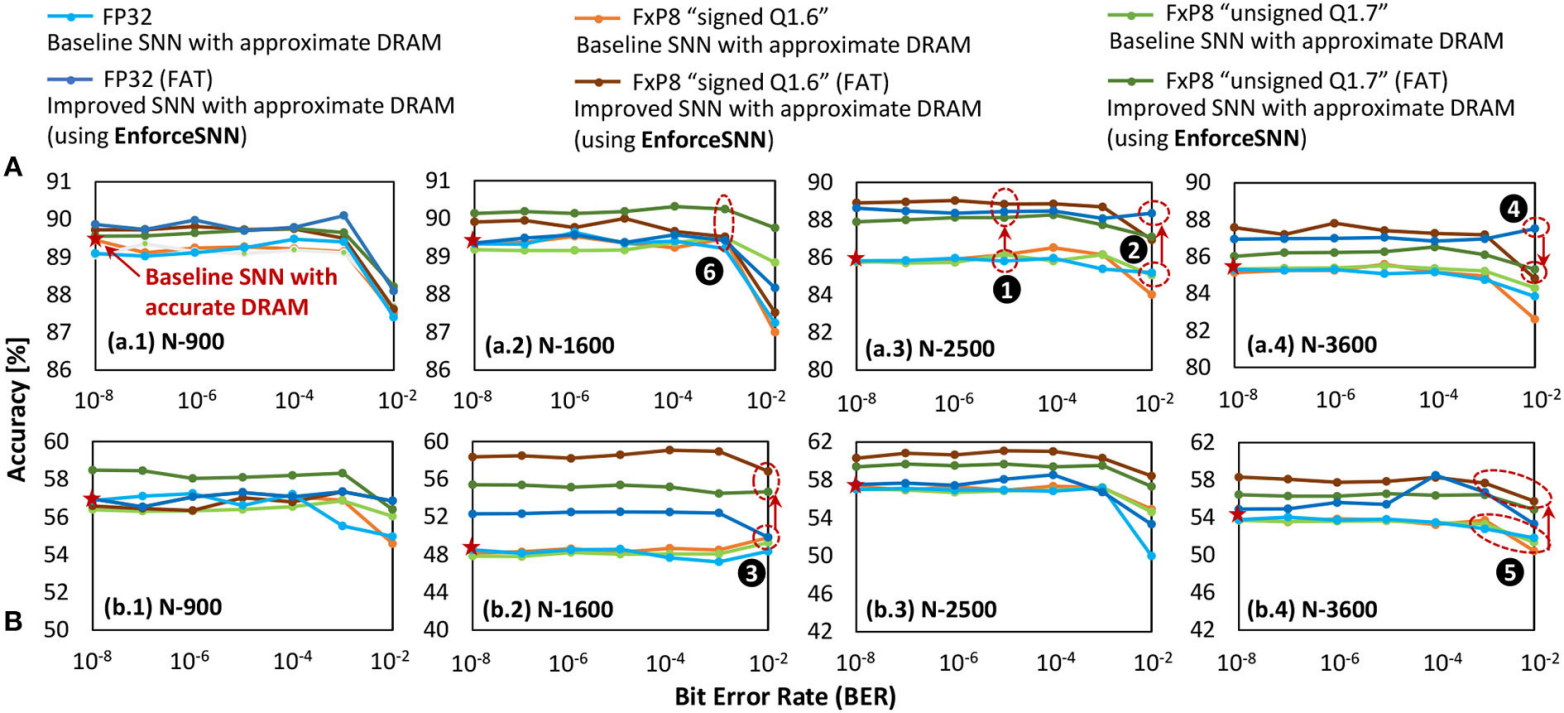
The accuracy of the baseline model with accurate and approximate DRAM, as well as the EnforceSNN-improved model with approximate DRAM for **(A)** MNIST and **(B)** Fashion MNIST datasets, across different precision levels, different BER values, and different network sizes.

Figure 11 shows the accuracy of the baseline model and the EnforceSNN-improved model with accurate and approximate DRAM across different BER values and precision levels, i.e., FP32 and FxP8 (“signed *Q*1.6” and “unsigned *Q*1.7”), network sizes, and workloads (i.e., the MNIST and Fashion MNIST datasets). In general, we observe that the baseline model with approximate DRAM achieves lower accuracy than the baseline model with accurate DRAM, and the accuracy decreases as the BER increases. These trends are observed across different weight precision levels, network sizes, and datasets. The reason is that the weights are changed (i.e., flipped) if they are stored in the faulty DRAM cells, and these weights are not trained to adapt to such bit flips. Therefore, the corresponding neuron behavior deteriorates from the expected behavior, hence decreasing accuracy. On the other hand, the EnforceSNN-improved model with approximate DRAM improves accuracy over the baseline model with accurate and approximate DRAM, across different BER values, network sizes, and datasets, as shown in ❶. We also observe that the EnforceSNN-improved model with approximate DRAM improves the accuracy over the baseline model with accurate and approximate DRAM, even in the high error rate case (i.e., *BER* = 10^−2^), as shown in ❷ for FP32 and ❸ for FxP8 weight precision levels. The reason is that our EnforceSNN incorporates the error profiles from the approximate DRAM across different BER values in the training process, which makes the SNN model adaptive to the presence of DRAM errors, thereby improving the SNN error tolerance. For the MNIST dataset, a high error rate (i.e., *BER* = 10^−2^) typically decreases the accuracy of the SNN-FxP8 more than the SNN-FP32, as shown in ❹. The reason is that the MNIST dataset has a narrow weight distribution in each class to represent its digit features, hence bit errors may change the weight values significantly in the FxP8 precision than the FP32 due to its shorter bit-width. As a result, the corresponding neuron behavior deteriorates from its ideal behavior, hence degrading accuracy. For the Fashion MNIST dataset, the SNN-FxP8 may achieve higher accuracy than the SNN-FP32 in some cases, as shown in ❺. The potential reason is the following. The Fashion MNIST dataset has relatively more complex features than the MNIST dataset, hence having a wider weight distribution in each class to represent its various features which may overlap with features from other classes (i.e., non-unique features). Then, the quantization removes these non-unique features by eliminating the less significant bits of the trained weights (i.e., like the denoising effect), and the retraining makes the quantized weights adaptive to bit flips, thereby leading to higher accuracy than the non-quantized ones. Furthermore, we also observe that the accuracy of the SNN-FxP8 starts showing notable degradation at a high error rate (i.e., *BER* = 10^−2^). For quantized models, in general, the “unsigned *Q*1.7” and “signed *Q*1.6” formats have similar trends and comparable accuracy as they represent similar weight values which differ only in the least significant fractional bit, thereby leading to similar neuron behavior and accuracy. These formats may have notable accuracy differences for some cases, such as after the retraining process, as shown by ❻. The possible reason is that these formats have different bit positions for the sign, integer, and fraction, thereby making the DRAM errors affect different weight bits and lead to different learning qualities during the respective fault-aware training.

In summary, our EnforceSNN maintains accuracy (i.e., no accuracy loss) as compared to the baseline with accurate DRAM when *BER* ≤ 10^−3^ across different datasets. Meanwhile, for higher BER values (i.e., 10^−3^<*BER* ≤ 10^−2^), our EnforceSNN still achieves higher accuracy than the baseline with accurate DRAM across different datasets. Therefore, these results show that *our EnforceSNN framework effectively improves the SNN error tolerance against DRAM errors with minimum retraining efforts*.

### 6.2. DRAM access energy savings and throughput improvements

**Figure 12:**
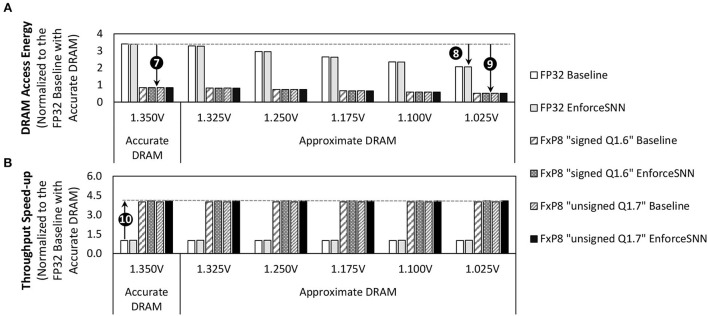
**(A)** The normalized DRAM access energy for an inference incurred by the baseline model and the EnforceSNN-improved model with accurate and approximate DRAM, and **(B)** the normalized speed-up of DRAM data throughput for an inference achieved by our EnforceSNN-improved model over the baseline model, across different *V*_*supply*_ values, different workloads (datasets), and different network sizes (N-900, N-1600, N-2500, and N-3600). These results are applicable for all network sizes. They are also applicable for both the MNIST and Fashion MNIST datasets, as these workloads have similar DRAM access energy, due to the same number of weights and number of DRAM accesses for an inference.

Figure 12A shows the normalized energy consumption of the DRAM accesses for an inference (i.e., inferring one input sample) required by the baseline model and the EnforceSNN-improved model with accurate and approximate DRAM, across different *V*_*supply*_ values, precision levels, network sizes, and workloads. We observe that different network sizes show similar normalized DRAM access energy, hence we only show a single figure representing the experimental results for all network sizes. For accurate DRAM cases across different network sizes, the baseline model achieves 75% DRAM energy saving when it employs the quantization technique, while our EnforceSNN-improved model achieves 75.1% DRAM energy saving due to the quantization and the proposed DRAM mapping policy, as shown in ❼. Meanwhile, the difference in these DRAM energy savings comes from the DRAM mapping policy. That is, our EnforceSNN optimizes the DRAM energy-per-access by maximizing the row buffer hits and the multi-bank burst feature, thereby having fewer row buffer conflicts than the baseline which only exploits the single-bank burst feature. For the FP32 precision across different network sizes, employing the approximate DRAM in the baseline model reduces the DRAM energy savings by up to 39.2% as compared to employing the accurate DRAM. Meanwhile, employing the approximate DRAM in the EnforceSNN-improved model reduces the DRAM energy savings by up to 39.5% as compared to employing the accurate DRAM, as shown in ❽. These energy savings come from the reduced DRAM energy-per-access due to the reduction of operational *V*_*supply*_. Moreover, the difference in energy savings between the baseline and our EnforceSNN also comes from the DRAM mapping policy. For the FxP8 precision (i.e., “signed *Q*1.6” and “unsigned *Q*1.7”) across different network sizes, employing the approximate DRAM in the baseline model reduces the DRAM energy savings by up to 84.8% over employing the accurate one. Meanwhile, employing the approximate DRAM in the EnforceSNN-improved model reduces the DRAM energy savings by up to 84.9% over employing the accurate one, as shown in ❾. These energy savings come from the reduced weight precision and the reduced DRAM energy-per-access due to *V*_*supply*_ reduction. Moreover, the difference in energy savings between the baseline and our EnforceSNN also comes from the DRAM mapping policy. Furthermore, we also observe that our EnforceSNN-improved model obtains 4.1x throughput speed-up over the baseline model across different *V*_*supply*_ values, workloads, and network sizes; refer to ❿ in Figure 12B. It is achieved through (1) the quantization technique which reduces the number of DRAM accesses, and (2) our proposed DRAM mapping policy which optimizes the DRAM latency-per-access by maximizing the row buffer hits and the multi-bank burst features. The results also show that the “unsigned *Q*1.7” and “signed *Q*1.6” achieve comparable DRAM access energy savings and throughput improvements since they employ the same bitwidth of weights, thereby having similar DRAM access behavior.

In summary, the results in Figure 12 indicate that *our EnforceSNN framework substantially reduces the DRAM access energy by employing the reduced-voltage approximate DRAM and our efficient DRAM mapping policy, while effectively improving the DRAM data throughput mainly due to the quantization*.

### 6.3. Model selection under design trade-offs

**Figure 13:**
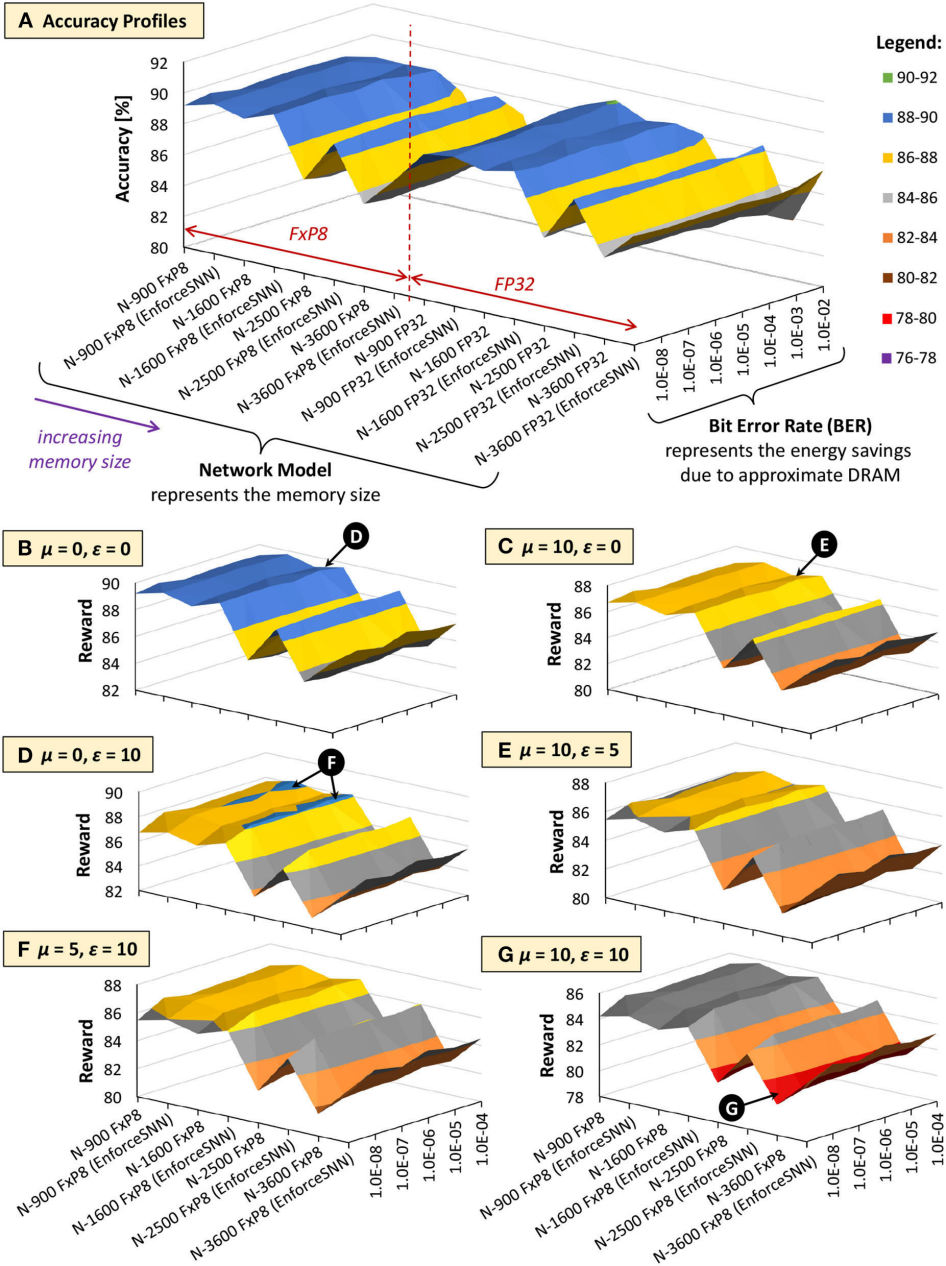
The trade-offs among accuracy, memory footprint, and energy consumption for the MNIST. **(A)** Accuracy profiles of SNN models. **(B–G)** Reward profiles of SNN models. The network sizes represent the memory sizes and the BER values represent the energy savings from approximate DRAM.

**Figure 14:**
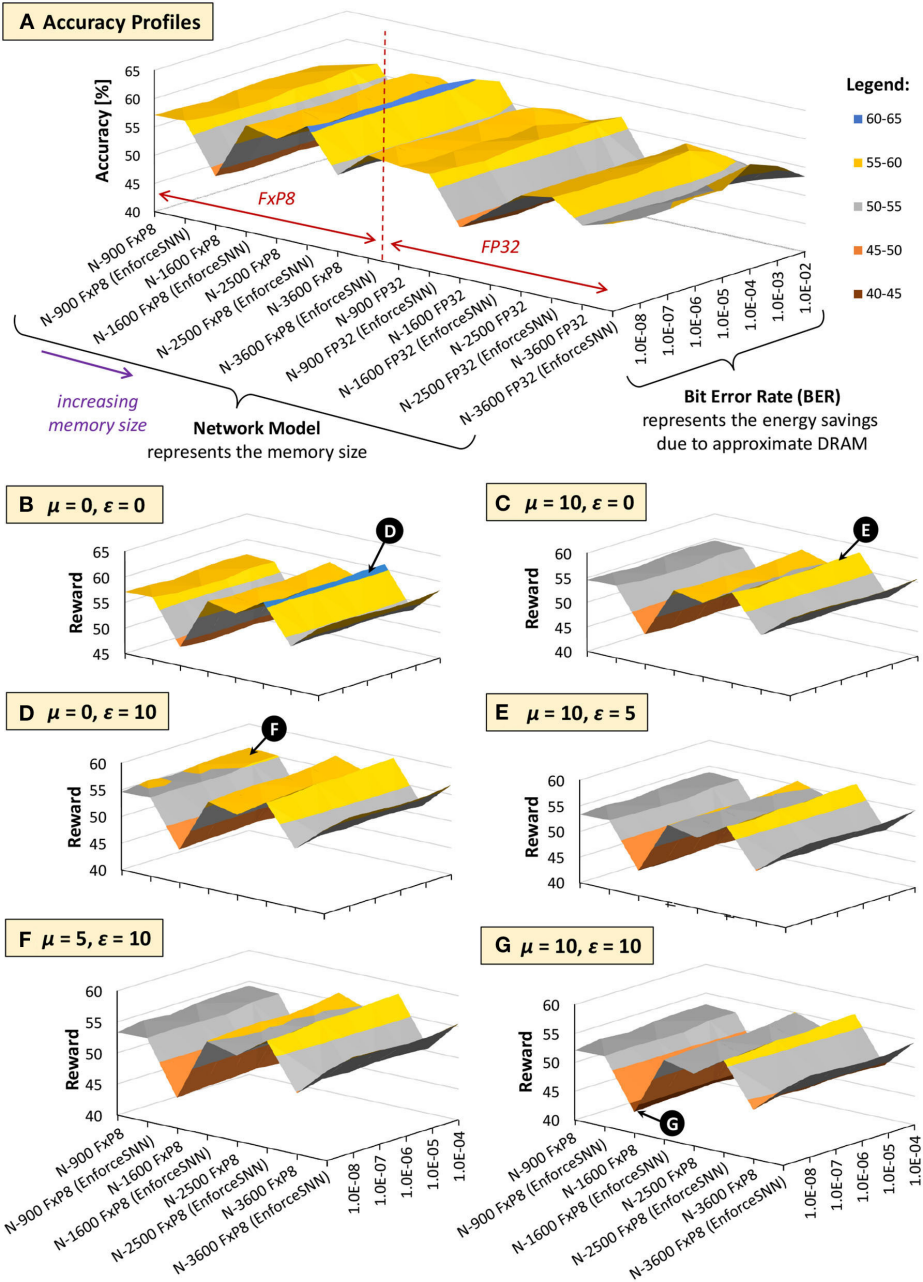
The trade-offs among accuracy, memory, and energy consumption for the Fashion MNIST. **(A)** Accuracy profiles of SNN models. **(B–G)** Reward profiles of SNN models. The network sizes represent the memory sizes, and the BER values represent the energy savings from approximate DRAM.

Figures 13, 14 show the results of the accuracy-memory-energy trade-offs for the MNIST and Fashion MNIST datasets, respectively. In this evaluation, the quantized models consider the FxP8 precision in “signed *Q*1.6” format. For the given SNN model candidates, we observe that the models that incur small memory size typically employ FxP8 precision, as shown in Figure 13A for the MNIST and Figure 14A for the Fashion MNIST. Considering that the accuracy of the FxP8-based models is comparable to the FP32-based models, we narrow down the candidates to only the FxP8-based models.

To analyze the design trade-offs, we explore the impact of different μ and ε values on the rewards. For instance, if we consider that the accuracy should have a higher priority than the memory and energy consumption, we set μ and ε low (e.g., μ = 0 and ε = 0). Meanwhile, if we consider that the memory should have a higher priority than the energy consumption, we set μ higher than ε (e.g., μ = 10 and ε = 0). For both cases, the highest reward is achieved by the EnforceSNN-improved N-1600 FxP8 for the MNIST and the EnforceSNN-improved N-2500 FxP8 for the Fashion MNIST under 10^−5^ error rate; refer to 🅓 for μ = 0 and ε = 0 cases, and refer to 🅔 for μ = 10 and ε = 0 cases. The reason is that these models employ our efficient FAT technique to improve their error tolerance, thereby leading to high accuracy under a high error rate. We also observe that having μ higher than ε makes the high rewards shift toward smaller models, as shown by 🅔 in Figure 13C. The reason is that a higher μ makes the small *m*_*norm*_ have a smaller impact on the reward reduction than the large *m*_*norm*_, thereby maintaining the high reward values. If the energy consumption should have a higher priority than the memory footprint, we set μ lower than ε (e.g., μ = 0 and ε = 10). The highest reward is achieved by the EnforceSNN-improved N-1600 FxP8 under 10^−5^ error rate for the MNIST and the EnforceSNN-improved N-2500 FxP8 under 10^−4^ error rate for the Fashion MNIST. In this case, we observe that high rewards are shifted toward models with smaller energy consumption (represented by higher BER); refer to 🅕 in Figures 13D, 14D. The reason is that a higher ε makes the small *E*_*norm*_ have a smaller impact on the reward reduction than the large *E*_*norm*_, thereby maintaining the high reward values. Furthermore, if the memory and energy consumption should have a higher priority than the accuracy, we set μ and ε high (e.g., μ = 10 and ε = 10). The highest reward is achieved by the EnforceSNN-improved N-1600 FxP8 under 10^−5^ error rate for the MNIST and the EnforceSNN-improved N-2500 FxP8 under 10^−4^ error rate for the Fashion MNIST. In this case, high rewards are shifted toward models with smaller memory and energy consumption (represented by high BER), but their overall rewards decrease as the values of μ and ε increase; refer to 🅖 in Figures 13E, 14E. The reason is that higher μ and ε jointly make the *m*_*norm*_ and *E*_*norm*_ decrease the reward. It means that if we want to significantly reduce the memory footprint and energy consumption, we have to accept more accuracy degradation.

In summary, the results in Figures 13, 14 show that *our EnforceSNN framework has an effective algorithm to trade off the accuracy, memory footprint, and energy consumption of the given SNN models*, thereby providing good applicability for diverse embedded applications with their respective constraints.

### 6.4. Optimization of the retraining costs

The conventional FAT for neural networks usually injects errors at an incremental rate during the retraining process from the minimum value to the maximum one for avoiding accuracy collapse (Koppula et al., 2019). Therefore, in this study, the conventional FAT considers *BER* = {10^−8^, 10^−7^, 10^−6^, …, 10^−2^}, while our efficient FAT in EnforceSNN only considers *BER* = {10^−4^, 10^−3^, 10^−2^} in the retraining process.

**Figure 15:**
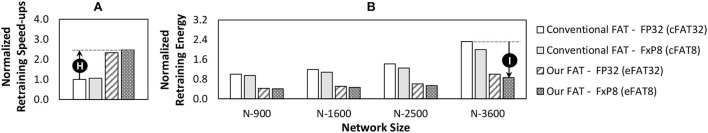
**(A)** The retraining speed-ups across different network sizes (i.e., N-900, N-1600, N-2500, and N-3600), and **(B)** the retraining energy for the MNIST, which are normalized to the conventional FAT with FP32 for a 900-neuron network. The results for the Fashion MNIST show similar trends to the MNIST since these workloads have similar DRAM access latency and energy due to the same number of weights and number of DRAM accesses for a training phase. Here, FxP8 represents both the “signed *Q*1.6” and “unsigned *Q*1.7” formats.

**Retraining Speed-ups:** The conventional FAT with FxP8 (cFAT8) obtains speed-up over the one with FP32 (cFAT32) by up to 1.16x and 1.14x for the MNIST and the Fashion MNIST respectively, since the cFAT8 employs quantized weights, thereby leading to a faster error injection and learning process. Meanwhile, our efficient FAT with FP32 (eFAT32) obtains a 2.33x speed-up over the cFAT32, since our eFAT32 has fewer iterations of the retraining process. Furthermore, we also observe that our efficient FAT with FxP8 (eFAT8) obtains more speed-up, i.e., by up to 2.71x for the MNIST and 2.65x for the Fashion MNIST as shown by 🅗 in Figure 15A, since our eFAT8 employs quantized weights in addition to fewer iterations of the retraining process.

**Retraining Energy Savings:** The cFAT8 achieves energy saving over the cFAT32 by up to 13.9% for the MNIST and 12% for the Fashion MNIST since the cFAT8 employs quantized weights which incur lower energy consumption during the error injection and learning process. Meanwhile, our eFAT32 achieves energy saving over the cFAT32 by 57.1%, as the eFAT32 performs fewer iterations of the retraining process as compared to the cFAT32. Our eFAT8 achieves further energy saving, i.e., by up to 63.1% for the MNIST and by up to 62.3% for the Fashion MNIST as shown by 🅘 in Figure 15B, since it employs quantized weights in addition to fewer iterations of the retraining process, thereby leading to a higher energy saving.

**Carbon Emission Reduction:** The retraining process also poses additional challenges that correspond to environmental concerns, i.e., carbon emission. Recent studies have highlighted that the carbon emission from neural network training should be minimized to prevent the increasing rates of natural disasters (Strubell et al., 2019, 2020). To estimate the carbon emission of neural network training, the study of Strubell et al. (2019) proposed Equation 5 and Equation 6. In these equations, *CO*_2_*e* denotes the estimated carbon (*CO*_2_) emission during the training, which is a function of the total power during the training (*p*_*t*_). Meanwhile, *t* is the training duration, *p*_*c*_ is the average power from all CPUs, *p*_*r*_ is the average power from all main memories (DRAMs), *p*_*g*_ is the average power from a GPU and *g* is the number of GPUs.


(5) $$\begin{array}{r} CO_{2}e=0.954\cdot p_{t} \end{array}$$

(6) $$\begin{array}{r} p_{t}=\frac{1.58\cdot t\left(p_{c}+p_{r}+g\cdot p_{g}\right)}{1000} \end{array}$$

These equations indicate that if we assume *p*_*c*_, *p*_*r*_, *p*_*g*_, and *g* are the same for different FAT techniques, then the difference will come from the training duration *t*. Therefore, our efficient FAT in EnforceSNN employs fewer iterations of the retraining process than the conventional FAT, thereby producing less carbon emission. Moreover, our EnforceSNN also reduces the operational power of the main memory (DRAM) through the reduced-voltage approximation approach, thereby further reducing the emission.

In summary, *our EnforceSNN framework effectively offers speed-up of retraining time, reduction of retraining energy, and less carbon emission than the conventional FAT technique*, thereby making it more friendly for our environments.

### 6.5. Further discussion

Previous studies that exploit the reduced-voltage DRAM concept mainly aim at improving the energy efficiency of mobile systems (Haj-Yahya et al., 2020), personal computing systems (Nabavi Larimi et al., 2021; Fabrício Filho et al., 2022), and server systems (David et al., 2011; Deng et al., 2011, 2012a,b; Nabavi Larimi et al., 2021). This concept is also employed for minimizing the energy consumption of deep neural networks (DNNs) (Koppula et al., 2019). Since SNNs have different data representation, computation models, and learning rules as compared to DNNs, our EnforceSNN provides a different framework with different techniques that are crafted specifically for improving the resilience and the energy efficiency of SNNs. Furthermore, the reduced-voltage DRAM is also used to generate noise (i.e., from DRAM errors) for obfuscating the intellectual property (IP) against security threats, such as IP stealing (Xu et al., 2020).

Our EnforceSNN framework can be put in the approximate computing field, especially in the context of the approximation for main memory through voltage scaling (Venkataramani et al., 2015; Mittal, 2016; Xu et al., 2016). Therefore, some of the techniques in our EnforceSNN are suitable for different domains outside SNNs: (1) DRAM voltage reduction for optimizing the DRAM access energy, (2) quantization for reducing the memory footprint, and (3) error-aware DRAM data mapping policy for minimizing the negative impact of DRAM errors on the data. These techniques are applicable for error-tolerant applications, such as image/video processing (e.g., data compression) and data analytic applications (e.g., data clustering).

## 7. Conclusion

We propose a novel EnforceSNN framework to achieve resilient and energy-efficient SNN inference considering reduced-voltage-based approximate DRAM, through weight quantization, error-aware DRAM mapping, SNN error-tolerance analysis, efficient error-aware SNN training, and effective SNN model selection. Our EnforceSNN achieves no accuracy loss for BER ≤ 10^−3^ with minimum retraining costs as compared to the baseline SNN with accurate DRAM while achieving up to 84.9% of DRAM energy saving and up to 4.1x speed-up of DRAM data throughput. In this manner, our study may enable efficient SNN inference for energy–constrained embedded devices like the Edge-AI.

## Data availability statement

Publicly available datasets were used in this study. These datasets can be found at the following sites: http://yann.lecun.com/exdb/mnist/ (MNIST), http://fashion-mnist.s3-website.eu-central-1.amazonaws.com/ (Fashion MNIST).

## Author contributions

RP, MH, and MS contributed to the conception of the framework. RP developed and implemented the framework, and wrote the first draft of the manuscript. RP and MH organized the experiments. MS supervised the research work. All authors contributed to manuscript revision, read, and approved the submitted version.

## Funding

This research is partly supported by the ASPIRE AARE Grant (S1561) on Towards Extreme Energy Efficiency through Cross-Layer Approximate Computing.

## Conflict of interest

The authors declare that the research was conducted in the absence of any commercial or financial relationships that could be construed as a potential conflict of interest.

## Publisher's note

All claims expressed in this article are solely those of the authors and do not necessarily represent those of their affiliated organizations, or those of the publisher, the editors and the reviewers. Any product that may be evaluated in this article, or claim that may be made by its manufacturer, is not guaranteed or endorsed by the publisher.

## Footnotes

^1^The research works for the unsupervised learning-based SNNs is still in the early stage and typically uses small datasets like the MNIST and the Fashion MNIST. We also adopt the same test conditions as used widely by the SNN research community.
